# Research on the bearing behavior of single pile in self-weight collapsible loess areas

**DOI:** 10.1371/journal.pone.0290878

**Published:** 2023-08-31

**Authors:** Denghui Gao, Kuanyao Zhao, Baohong Ma, Zhiping Han, Jifei Fan

**Affiliations:** 1 College of Architecture and Civil Engineering, Huanghuai University, Zhumadian, Henan, China; 2 Gansu Provincial Highway Aviation Tourism Investment Group Corporation Limited, Lanzhou, China; 3 Shanghai Baoye Group Corporation Limited, Shanghai, China; 4 School of Civil Engineering, Lanzhou University of Technology, Lanzhou, China; Balochistan University of Information Technology Engineering and Management Sciences, PAKISTAN

## Abstract

The negative skin frictional caused by loess collapse will decrease the bearing capacity of single pile, which is essential to the design of pile foundations in loess areas. In this study, a method for estimating the subsidence of soil layer at any depth is firstly proposed based on the total self-weight collapse value. Secondly, a new load transfer constitutive model for pile-soil interface is developed, which considers the nonlinear stress-strain relationship and the ultimate shear strength of soil. Then, a load transfer calculation model for pile foundation is established, which can calculate the pile axial force, the pile skin frictional, neutral point position and the settlement of a single pile. The calculation results are compared with the test data that obtained from a pile foundation on-site immersion test and the effectiveness of the calculation method is verified well. This calculation method may be useful for designing pile foundations in collapsible loess regions.

## Introduction

Loess is extensively distributed around the world. In northwest China, loess is widely distributed and has a large thickness, which is characterized by collapsibility and water sensitivity [1]. Pile foundation is a common foundation type in collapsible loess areas, which can penetrate the soft or loose soil layers and transmit the structural load to competent soil layers, and providing a great bearing capacity by positive shaft resistance and pile tip resistance. But, when the settlement of soil surrounding a pile is greater than that of the pile, the positive shaft resistance will transform into the negative skin friction, which decreases the bearing capacity of single pile and increases the compressive stress in the pile shaft [2]. Due to the wetting-sensitive of unsaturated loess, the collapsible loess may products a significant subsidence after water immersion under the combined action of soil overburden self-weight pressure and additional pressure. Then, the pile may be subjected to a significant negative skin friction and the pile capacity will be reduced or perhaps catastrophic failure [3]. Hence, it is essential to calculate the bearing behavior of single pile with the negative skin friction in loess area.

The negative skin friction of single pile in loess area is caused by the collapsible deformation of loess after water immersion. Loess is widespread continental sediment which posses a metastable structure featured by open fabric and inter-particle bonding [4]. The cementation materials of inter-particle bonding in natural loess are always carbonate and clays [5], and will be destroyed under the combined action of soil self-weight pressure and water, undergo a significant collapsible deformation. The research on the collapsible deformation calculation method of loess is mainly based on the experimental rules obtained from oedometer tests and triaxial tests, and corrected or verified with the test result of on-site immersion to ensure the applicability of the calculation method. The constitutive model of loess is established based on the triaxial test rules that can describe the stress-strain relationship of loess with different humidity under complex stress state, which is suitable for the finite element calculation of collapsible deformation [6–10]. However, many model parameters and theoretical complexity limit its wide application. The most common collapse deformation calculation method is to calculate the summation deformation of all collapsible loess layers by using the collapsibility coefficient [11]. The collapsible coefficient can be obtained by using oedometer tests or triaxial tests [12–14]. The total collapse settlement can be calculated accurately by multiplying the summation deformation of all layers and the correction coefficient of regional collapsibility that obtained from the field immersion test [1]. However, due to the complexity of water vapor transport in soil layers [15] and the constraining effect of surrounding soil [16], the distribution rules of stress in the large thickness self-weight collapsible loess is relatively complex. The subsidence calculation of soil layer at any depth cannot be calculated accurately, which makes it difficult to calculate the relative displacement of pile-soil.

The negative skin friction of single pile caused by loess collapse deformation is usually based on empirical value, and there is no a complete calculation method. The empirical value of negative skin friction is usually determined according to the pile foundation on-site immersion test [17–19], model test [2, 20, 21] and centrifugal test [22, 23]. The empirical value is too conservative to reflect the distribution rule of negative friction along the pile length. It is necessary to establish a load transfer function that can reflect the distribution rule of the pile negative skin friction. The pile–soil load transfer function can be obtained from the soil–structural material interface tests. However, the load transfer function, such as the hyperbolic interface model [24], the load transfer model considering shaft resistance softening [25] and the linearly elastic-perfectly plastic model [26], which are established based on the soil-structure interface tests that mainly focused on clay soil [27], sandy soil [20], and cemented soil [28], the interface tests few focused on loess. Hence, it is difficult to establish the pile-loess load transfer function based on the interface tests. The load acting on pile shaft is transferred to the soil in the form of shear stress through a single pile, causing shear deformation of the soil around the pile in a certain range [29]. Hence, it is a convenient method to establish the load transfer function according to the mechanical characteristics of the soil around the pile, namely, the shear displacement method [30]. The research on the mechanical characteristics of loess is sufficient, which is available to establish the load transfer function of pile-loess.

Accordingly, this paper aims to research the bearing behavior of single pile with the negative skin friction in loess area. To achieve this objective, the subsidence calculation method of loess layer at any depth based on the total collapse settlement is proposed, and the calculation of pile-loess relative displacement under collapsible deformation condition is realized. A new load transfer constitutive model based on the strength and deformation of the soil around the pile is developed, which considers the nonlinear stress-strain relationship and the ultimate shear strength of soil. According to the static equilibrium of pile segment, the load transfer calculation model is established by using the load transfer constitutive model and pile-soil relative displacement, which can realize the calculation of single pile bearing behavior under the collapsible deformation condition.

## Subsidence calculation of soil layer at any depth

### Calculation of the total self-weight collapse value

The loess standard [31] provides a relatively simple calculation method for self-weight collapsible settlement. The total self-weight collapse value can be obtained by using the correction coefficient of regional collapsibility, the self-weight collapsible coefficient and the thickness of self-weight collapsible soil layer. In this study, we used this calculation method to estimate the total self-weight collapse value:

The self-weight collapsible coefficient *δ*_*zs*_ is calculated as follows:

$$\delta_{zs}=\frac{h_{z}-h_{z}^{'}}{h}$$ (1)

Where, *h*_*z*_ is the stabilized height of specimen with natural humidity and structure under the action of overburden self-weight pressure (mm); $h_{z}^{'}$ is the stabilized height of saturated specimen under the same pressure after immersion (mm); and *h* is the initial height of specimen (mm).

The total self-weight collapse value *s*_0_ is calculated as follows:

$$s_{0}=\beta_{0}\sum_{i=1}^{n}\delta_{zsi}h_{i}$$ (2)

Where, *δ*_*zsi*_ is the self-weight collapsible coefficient of the *i*th layer soil, *h*_*i*_ is the thickness of the *i*th layer soil, and *β*_0_ is the correction coefficient of regional collapsibility.

The loess layer with the self-weight collapsible coefficient *δ*_*zs*_ less than 0.015 is regarded as the non-collapsible soil layer under self-weight stress. The deformation of non-collapsible soil layer is not included in the total self-weight collapse deformation and the depth of non-collapsible layer is the settlement calculation lower limit depth.

### Calculation of stratified soil subsidence

The total self-weight collapse value can be obtained by using the correction coefficient of regional collapsibility, the self-weight collapsible coefficient and the thickness of self-weight collapsible soil layer. But, there is a large deviation between the subsidence calculation value and the measured value of stratified soil in the on-site immersion test [32]. It can be seen from the curve of subsidence with depth that the subsidence variation law of stratified soil is similar to the Boussinesq’s vertical displacement solution [33–34]. Therefore, we can deduce the corresponding concentrated force *F* according to the total self-weight collapse value, and use the Boussinesq’s vertical displacement solution to calculate the stratified soil subsidence.

When the self-weight stress is small, the self-weight collapsible coefficient will be less than 0.015, and the collapsible deformation caused by soaking under self-weight stress can be ignored. The self-weight stress increases with the burial depth, and the self-weight collapsible coefficient gradually increases. When the self-weight collapsible coefficient is equal to 0.015, the collapsible deformation should be calculated, and the burial depth of the soil layer is the initial self-weight collapsible depth *h*_0_. The initial self-weight collapsible depth *h*_0_ is the action position of the concentrated force *F* in the Boussinesq’s solution. Above this depth, the self-weight collapse deformation of soil layer was 0, and stratified soil subsidence is equal to the total self-weight collapse value *s*_0_. The subsidence of non-collapsible soil layers caused by soaking under self-weight stress is also very small, which can be ignored compared with the total settlement. Hence, the burial depth of non-collapsible soil layer is the lower limit depth *h*_*e*_ for the calculation of collapsible deformation [31]. The subsidence of soil layer at or below this depth was 0.

Under the action of the concentrated force *F*, the expression of Boussinesq’s vertical displacement at an arbitrary point is modified as follows:

$$s{\left(z\right)}^{'}=\frac{F(1+\mu )}{2\pi E}[\frac{{\left(z-h_{0}\right)}^{2}}{{\left(\sqrt{{\left(z-h_{0}\right)}^{2}+R^{2}}\right)}^{3}}+\frac{2(1-\mu )}{\sqrt{{\left(z-h_{0}\right)}^{2}+R^{2}}}]$$ (3)

Where, *μ* is Poisson’s ratio of the soil and *E* is the elastic modulus of the soil. To prevent *s*(*z*)’ from approaching infinity, the horizontal distance between the calculated point and the action point of the concentrated force *R* is set to be 0.8 m, which is the diameter of the pile.

The subsidence of soil layer at *z* = *h*_0_ is *s*_0_, the concentrated force *F* is deduced as follows:

$$F=\frac{s_{0}\pi ER}{\left(1-\mu )(1+\mu \right)}$$ (4)

Subsequently, Eq (4) is substituted into Eq (3) to obtain Eq (5), which can be used for calculating the subsidence of soil layer at any depth based on the total self-weight collapse value:

$$s{\left(z\right)}^{'}=\frac{s_{0}R}{2(1-\mu )}[\frac{{\left(z-h_{0}\right)}^{2}}{{\left(\sqrt{{\left(z-h_{0}\right)}^{2}+R^{2}}\right)}^{3}}+\frac{2(1-\mu )}{\sqrt{{\left(z-h_{0}\right)}^{2}+R^{2}}}]$$ (5)

Because of the subsidence of soil layer at lower limit depth *h*_*e*_ is assumed to be 0. So, the subsidence at the depth *z*∈[*h*_0_,*h*_*e*_] is modified as follows:

$$s(z)=s{\left(z\right)}^{'}-s{\left(h_{e}\right)}^{'}$$ (6)

When the depth of soil layer *z*≤*h*_0_, its subsidence is *s*_0_, which can be calculated using Eq (2). When the depth of soil layer *z*>*h*_*e*_, this soil layer subsidence is 0. When the depth of soil layer z between *h*_0_ and *h*_*e*_, the subsidence is calculated using Eq (6). Thus, the subsidence of soil surrounding a pile at any depth can be calculated.

## Pile–soil load transfer constitutive model

### Principle and existing problems of shear displacement method

**Fig 1:**
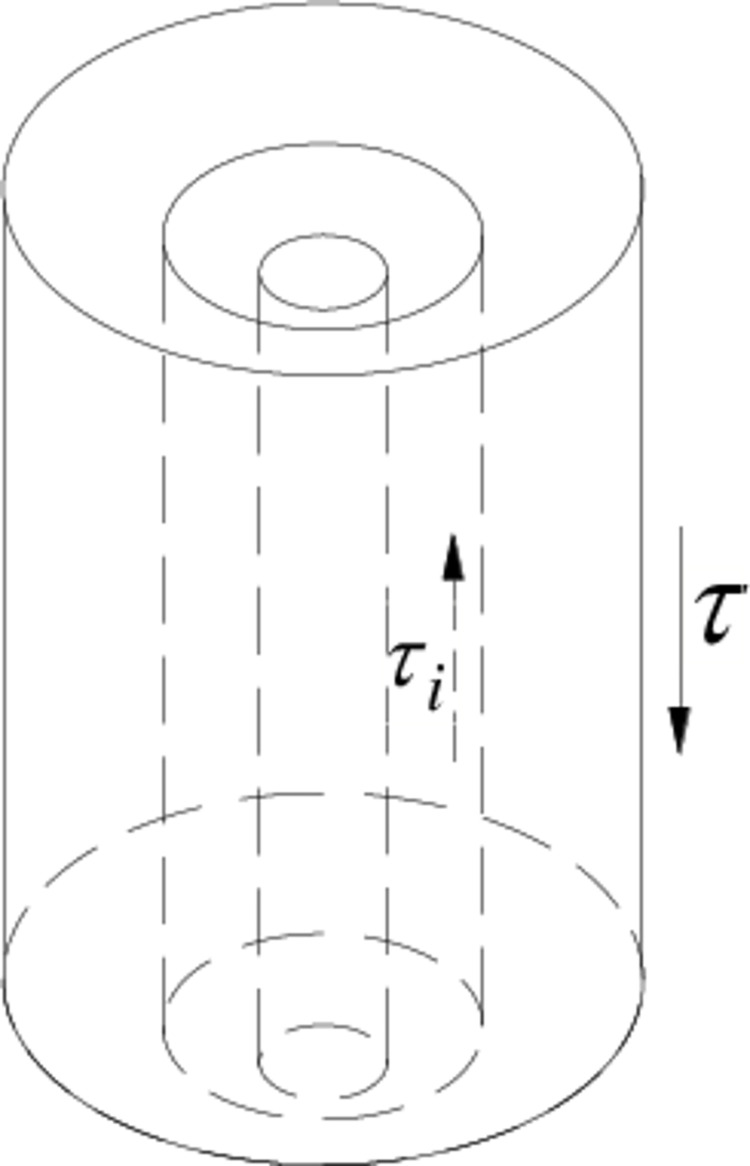
The deformation schematic diagram of soil around pile.

The shear displacement method assumes that the upper load is transferred to the soil in the form of shear stress through a single pile, causing shear deformation of the soil around the pile in a certain range [29], as shown in Fig 1.

**Fig 2:**
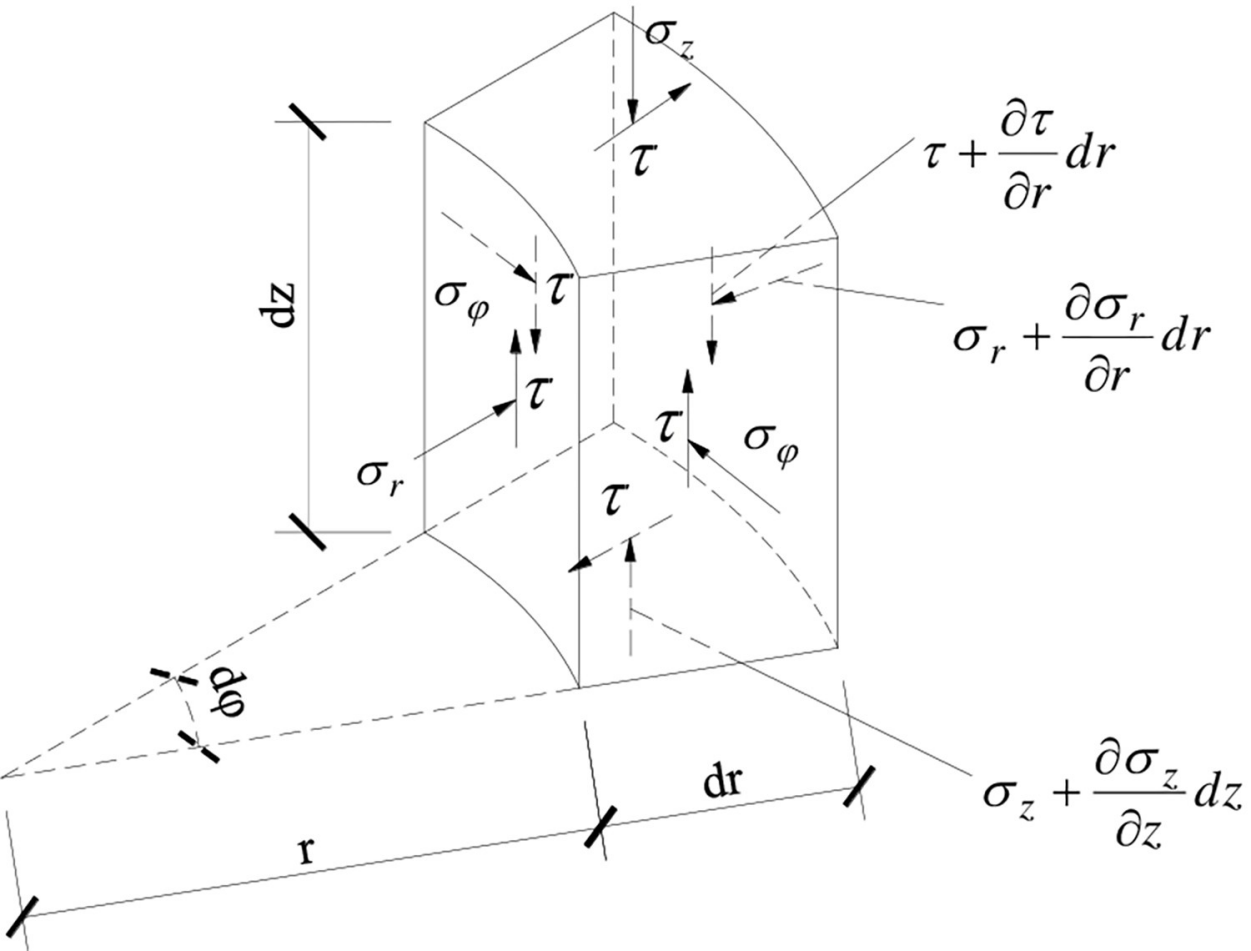
The force analysis of soil micro-unit.

The stress analysis on the micro-unit of soil around the pile is carried out, as shown in Fig 2. According to the vertical static equilibrium differential equation of the micro-unit, the relationship between the pile skin friction and the shear stress of soil can be obtained as follows:

$$\tau =\frac{\tau_{0}\cdot r_{0}}{r}$$ (7)

Where, *τ*_0_ and *r*_0_ are the pile skin friction and the radius of the pile, respectively. *τ* is the shear stress acting on the soil micro-unit, and *r* is the distance between soil micro-unit and pile axis.

According to the geometric equation of elasticity theory, and the radial deformation of the micro-unit can be omitted, then the expression of shear strain can be obtained as follows:

$$\gamma =\frac{\partial \Delta s}{\partial r}$$ (8)

Where, Δ*s* is the vertical relative displacement of pile-soil at the distance *r* from the pile axis.

According to the generalized Hooke’s law, the constitutive relationship between shear stress and shear strain is obtained as follows:

$$\gamma =\frac{\tau }{G_{s}}$$ (9)

Substituting Eqs (8) and (9) into Eq (7) for solution, the load transfer function of shear displacement method can be obtained as follows:

$$\tau_{0}=\frac{G_{s}\Delta s}{r_{0}\ln\frac{r_{m}}{r_{0}}}$$ (10)

Where, *G*_*s*_ is the shear modulus of the soil, and *r*_*m*_ is the maximum influence radius of pile skin friction on the soil around the pile, its recommended value is *r*_*m*_ = 10*r*_0_ [35].

Because the load transfer function of shear displacement method is derived on the basis of elastic theory, it can be seen from Eq (10) that the load transfer between piles and soil is a linear function. The greater the relative displacement of pile-soil, the greater the pile skin friction, which obviously inconsistent with the load transfer law of pile-soil measured on site. The reason is that the nonlinearity of the stress-strain relationship of soil is not fully considered during the theoretical derivation. The shear modulus of the soil changes with the shear strain, which is not a fixed value.

### New load transfer model based on shear displacement method

**Fig 3:**
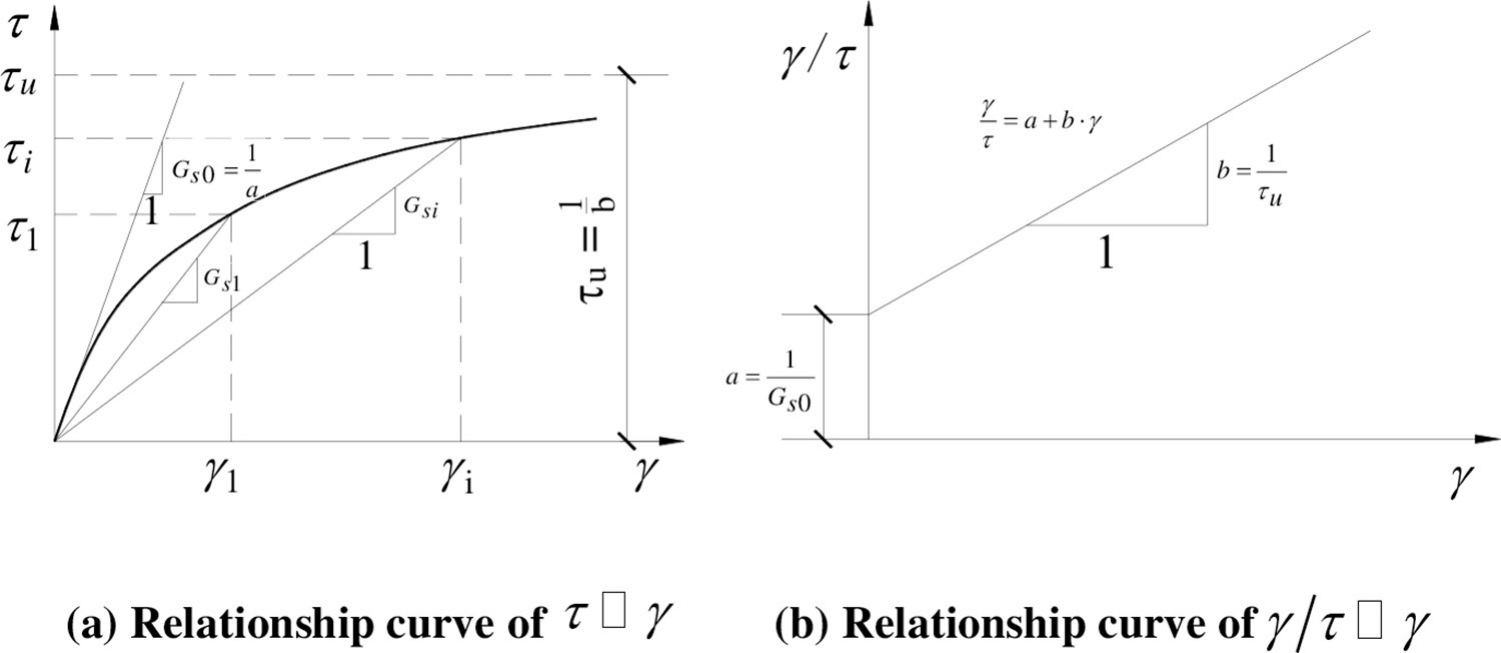
Schematic diagram of shear stress-strain relationship curve. (a) Relationship curve of *τ*~*γ* (b) Relationship curve of *γ*/*τ*~*γ*.

The stress-strain curve of natural loess obtained by the triaxial shear test is approximately hyperbolic [4], as shown in Fig 3(A), which can be expressed as follows:

$$\tau =\frac{\gamma }{a+b\cdot \gamma }$$ (11)

Eq (11) can be transformed into Eq (12), which is also the expression of shear modulus versus with shear strain.


$$\frac{\tau }{\gamma }=G_{s}=\frac{1}{a+b\cdot \gamma }$$ (12)

Where, *a* and *b* are test parameters of soil, with a physical meaning for the inverse of the initial shear modulus *G*_*s*0_ and the inverse of the ultimate shear strength *τ*_*u*_, respectively. In the *γ*/*τ*~*γ* coordinates, these test parameters can be obtained by linear fitting, as shown in Fig 3(B).

Parameters *a* and *b* are related to the stress state of soil, the parameter *b* can be calculated using the method in Duncan-Chang model [36], the calculation formula is as follows:

$$G_{s0}=\frac{1}{a}=Kp_{atm}{\left(\frac{\sigma_{3}}{p_{atm}}\right)}^{n},$$ (13)

Where, *K* and *n* are the parameters of the model, and *p*_*atm*_ is the standard atmospheric pressure. According to the actual shear direction of soil, the confining pressure *σ*_3_ is regarded as the overburden self-weight stress of soil in saturated state, namely, *σ*_3_ = *γ*_*sat*_*z*.

The inverse of parameter *b* is the ultimate shear strength of the soil:

$$\tau_{u}=\frac{1}{b}=c+K_{0}\sigma_{z}\tan\varphi$$ (14)

Where, *c* and *φ* are the cohesion and friction angles of saturated soil, respectively. For the soil with obvious anisotropic characteristics, the strength parameters can be measured using triaxial shear tests according to the actual shear direction, or using the reduced strength parameters.

**Fig 4:**
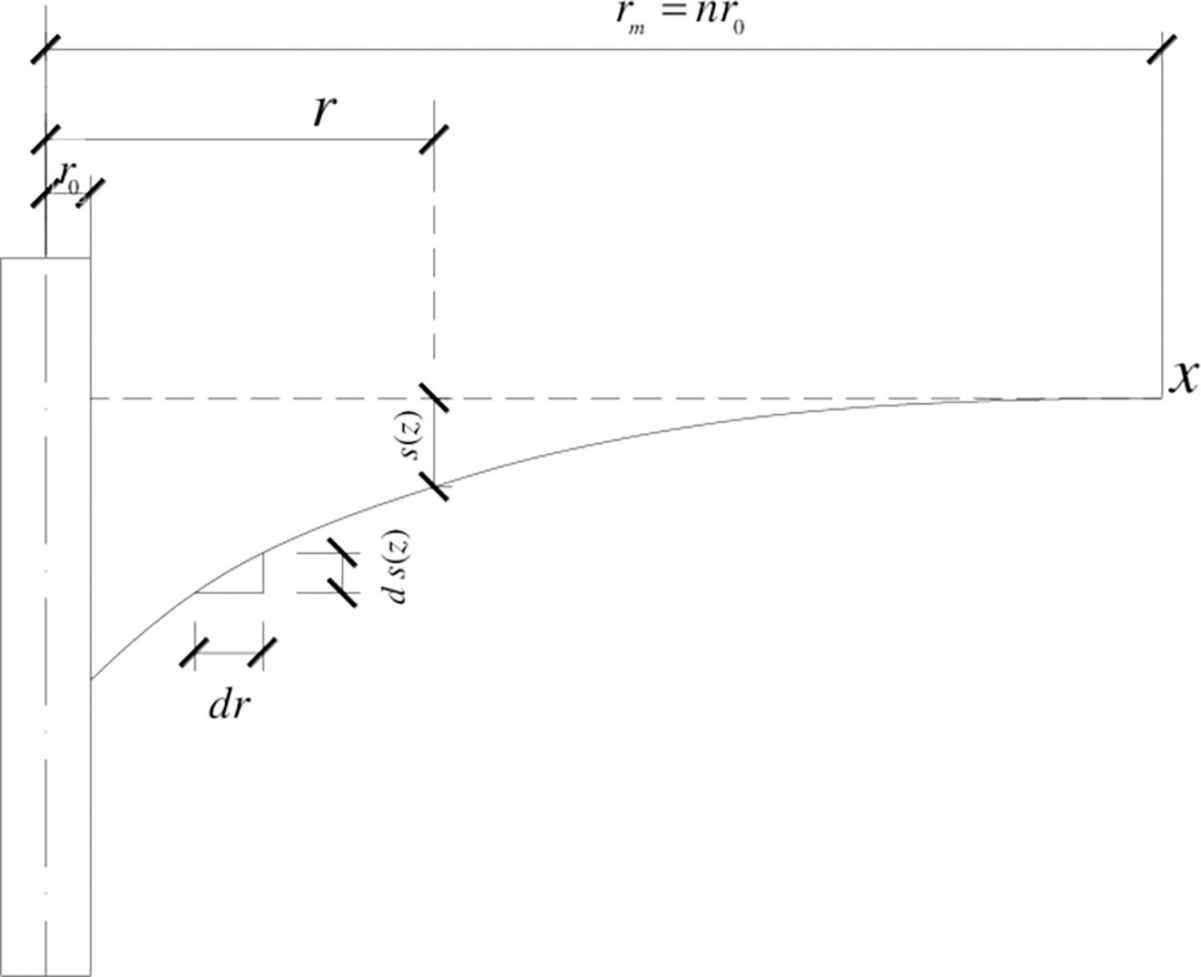
The variation of pile-soil relative vertical displacement in the radial.

The variation of soil vertical displacement in the radial is nonlinear, as shown in Fig 4. The model for pile-soil vertical relatived is placement Δ*s*(*z*) versus radial distance *γ* is usually described by hyperbola and parabola [37]. This assumption is appropriate for large scale on-site immersion test, but not applicable for local immersion condition.

The relational expression is defined as follows:

$$\Delta s(z)=\frac{\alpha }{r}+\beta$$ (15)

Where, the range of *r* is [*r*_0_,*r*_*m*_], the range of Δ*s*(*z*) is [0,Δ*S*(*z*)], Δ*S*(*z*) is the maximum pile–soil relative displacement at depth *z*, and *α* and *β* are the parameters of the curve.

The boundary conditions are determined as follows:

$$0=\frac{\alpha }{r_{m}}+\beta$$ (16)

$$\Delta S(z)=\frac{\alpha }{r_{0}}+\beta$$ (17)

The parameters *α* and *β* can be obtained by solving simultaneous Eqs (16) and (17).


$$\alpha =\Delta S(z)/\left(\frac{1}{r_{0}}-\frac{1}{r_{m}}\right)$$ (18)

$$\beta =\Delta S(z)/\left(1-\frac{r_{m}}{r_{0}}\right)$$ (19)

Hence, the calculation of shear strain in Eq (8) can be obtained as follows:

$$\frac{\partial \Delta s(z)}{\partial r}=-\frac{\alpha }{r^{2}}=\frac{\Delta S(z)}{r^{2}\cdot (\frac{1}{r_{m}}-\frac{1}{r_{0}})}=-\gamma$$ (20)

The calculation equation of soil shear modulus in the radial under different pile-soil relative displacements can be obtained by substituting Eq (20) into Eq (12) as follows:

$$G_{s}=\frac{1}{a+\frac{b\cdot \alpha }{r^{2}}}$$ (21)

Substituting Eq (21) into Eq (9), and the constitutive relationship between shear stress and shear strain can be transformed into the following form.


$$\gamma =\frac{\tau }{G_{s}}=\frac{\tau_{0}\cdot r_{0}}{r}\cdot (a+\frac{b\cdot \alpha }{r^{2}})$$ (22)

Hence, the new load transfer model can be obtained by substituting Eq (22) into Eq (8):

$$\tau_{0}=\frac{\Delta S(z)}{r_{0}\cdot [a\cdot \ln(\frac{r_{m}}{r_{0}})+\frac{b\cdot \Delta S(z)}{2}\cdot (\frac{1}{r_{0}}+\frac{1}{r_{m}})]}$$ (23)

Finally, the new load transfer model considering the nonlinear deformation of the soil around the pile is established. Compared with Eq (10), the pile skin friction in the new load transfer model approached an extreme value when the pile-soil relative displacement is large, which is consistent with the load transfer law of pile-soil measured on site.

## Calculation method of single pile bearing behavior

**Fig 5:**
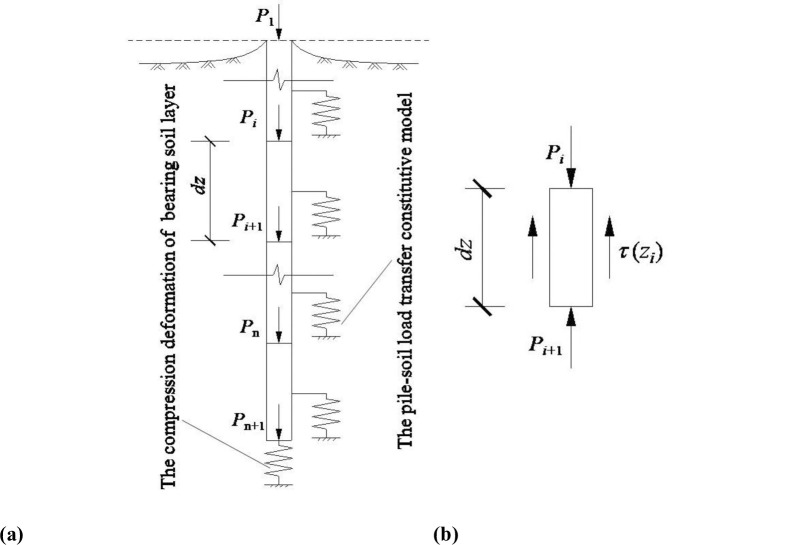
The load transfer model of single pile. (a) Schematic diagram of pile bearing behavior (b) Stress analysis of the *i*th pile segment.

The load transfer model of single pile is shown in Fig 5. The pile is divided into *n* segments along the length direction, and the calculation equation can be built according to the static equilibrium of each pile segment.

### Subsidence calculation of pile segment at any depth

As shown in Fig 5(A), the pile is divided into *n* segments. The subsidence of pile segment at depth *z* is composed of two parts: namely, the pile tip subsidence and the summation of the compression deformation of pile segments below this depth. The subsidence of pile segment at any depth can be calculated as follows:

$$s_{p}(z_{i})=S_{b}+ds_{n}+ds_{n-1}+\cdots +ds_{i+1}$$ (24)

Where, *s*_*p*_(*Z*_*i*_) is the subsidence of the *i*th pile segment, *S*_*b*_ is the pile tip subsidence, *ds*_*i*_ is the compression deformation of the *i*th pile segment.

It is assumed that the pile segment compression process is in the elastic stage. The forces acting on the pile segment include: the pile skin friction, the axial force acting on the upper and lower interfaces, as shown in Fig 5(B). The compression deformation of the *i*th pile segment can be calculated according to the following equation:

$$ds_{i}=\frac{P_{i}+P_{i+1}}{2A_{p}E_{p}}dz$$ (25)

Where, *A*_*p*_ is the cross-sectional area of pile shaft and *E*_*p*_ is the elastic modulus of pile material.

The pile tip subsidence is given by the Boussinesq’s solution [38].


$$S_{b}=\frac{P_{n+1}\cdot (1-\mu_{s})}{4r_{0}\cdot G_{s}}$$ (26)

Where, *P*_*n*+1_ is the axial force at the pile tip; *μ*_*s*_ and *G*_*s*_ are Poisson’s ratio and the shear modulus of the bearing soil layer, respectively.

The shear modulus of the bearing soil layer *G*_*s*_ can be obtained in accordance with its relationship with the compressive modulus *E*_*s*_ as follows [30]:

$$G_{s}=\frac{E_{s}}{2(1+\mu_{s})}(1-\frac{2\mu_{s}{}^{2}}{1-\mu_{s}})$$ (27)

Hence, the subsidence of pile segment at any depth can be calculated with the pile tip subsidence.

### Calculation of the pile axial force at any depth

According to the static equilibrium of pile segment in Fig 5(B), the following equation can be established:

$$P_{i}=P_{i+1}+\tau_{0}(z_{i})\times 2\pi r_{0}\times dz$$ (28)

Where, *τ*_0_(*z*_*i*_) is the *i*th pile segment skin frictional, which can be calculated using Eq (23).

The subsidence of pile and soil can be calculated by Eqs (24) and (6), respectively. The pile–soil relative vertical displacement Δ*S*(*z*_*i*_) in Eq (23) can be calculated using the following equation:

$$\Delta S(z_{i})=s_{p}(z_{i})-s(z_{i})$$ (29)

The axial force of the pile shaft at any depth can be calculated by using Eq (26) and Eq (28), when the pile tip subsidence is given. The axial force calculation equation of pile shaft at any depth is as follows:

$$P_{i}=\frac{4r_{0}\cdot G_{s}\cdot S_{b}}{1-\mu_{s}}+2\pi r_{0}\times \sum_{i=1}^{n}\tau_{0}(z_{i})\times dz$$ (30)

The solution can be obtained by following an iterative procedure. Firstly, assume a pile tip subsidence initial value *S*_*b*_, which is also considered as the subsidence of the *n*th pile segment. The axial force acting on the lower interface of the *n*th pile segment *P*_*n*+1_ can be calculated by substituting *S*_*b*_ into Eq (26). Secondly, the pile skin frictional of the *n*th pile segment *τ*_0_(*z*_*n*_) can be calculated as follows: the subsidence of the *n*th pile segment is *S*_*b*_, and the subsidence of soil around the pile tip *s*(*z*_*n*_) can be calculated by using Eq (6), thus, the pile-soil relative displacement of the *n*th pile segment Δ*S*(*z*_*n*_) can be calculated by substituting *S*_*b*_ and *s*(*z*_*n*_) into Eq (29), and the pile skin frictional of the *n*th pile segment *τ*_0_(*z*_*n*_) can be calculated by substituting Δ*S*(*z*_*n*_) into Eq (23). Thirdly, the axial force acting on the upper interface of the *n*th pile segment *P*_*n*_ can be calculated by substituting *P*_*n*+1_ and *τ*_0_(*z*_*n*_) into Eq (28), and the compression deformation of the *n*th pile segment *ds*_*n*_ can be calculated by substituting *P*_*n*_ and *P*_*n*+1_ into Eq (25). Hence, the subsidence of the (*n*-1)th pile segment can be calculated by substituting *ds*_*n*_ into Eq (24). Finally, the axial force acting on the upper interface of the 1th pile segment *P*_1_ can be calculated through successive iterations. The *P*_1_ value is the load value acting on the pile head, which is known. Adjust the pile tip subsidence initial value *S*_*b*_, repeat the procedure until convergence is achieved normally (The *P*_1_ calculated value is consistent with the pile head action load).

### Calculation of the pile skin frictional and neutral point position

The pile tip subsidence value *S*_*b*_ can be obtained when the iteration convergence. According to the iterative procedure, once the pile tip subsidence value *S*_*b*_ is determined, the lower interface of the *n*th pile segment *P*_*n*+1_ can be calculated by substituting *S*_*b*_ into Eq (26). Besides, the subsidence of soil around the pile tip *s*(*z*_*n*_) can be calculated by using Eq (6), the relative displacement of pile-soil Δ*S*(*z*_*n*_) can be calculated by substituting *S*_*b*_ and *s*(*z*_*n*_) into Eq (29). Hence, the pile skin frictional of the *n*th pile segment *τ*_0_(*z*_*n*_) can be calculated by substituting Δ*S*(*z*_*n*_) into Eq (23). Furthermore, the axial force acting on the upper interface of the *n*th pile segment *P*_*n*_ can be calculated by substituting *P*_*n*+1_ and *τ*_0_(*z*_*n*_) into Eq (28), and the compression deformation of the *n*th pile segment *ds*_*n*_ can be calculated by substituting *P*_*n*_ and *P*_*n*+1_ into Eq (25). Then, the subsidence of the (*n*-1)th pile segment *s*_*p*_(*z*_*i*_) can be calculated by substituting *ds*_*n*_ into Eq (24). The relative displacement of pile-soil Δ*S*(*z*_*i*_) and the pile skin frictional of the *n*th pile segment *τ*_0_(*z*_*i*_) can be calculated through successive iterations by Eq (29) and Eq (23), respectively.

The neutral point position is the depth where the relative displacement of pile-soil is 0. The subsidence of the pile *s*_*p*_(*z*_*i*_) and the subsidence of the soil around the pile *s*(*z*_*i*_) can be calculated by Eq (24) and Eq (6), respectively. When the subsidence of pile and soil is equal, namely, Δ*S*(*z*_*i*_) = 0 in Eq (29), the corresponding *z* value is the depth of the neutral point position.

## Verification of the calculation method

To verify the reasonableness of the calculation method, the calculated results are compared with the test data of pile foundation on-site immersion test, which is carried out on the self-weight collapsible loess site in Weinan, Shaanxi, China [34]. The lower limit depth for the calculation of collapsible deformation is 33m in this site. The test pile is 60m long and 0.8m in diameter, which is a bored pile with the pile shaft concrete strength grade of C35. The elastic modulus of pile shaft material is taken as 31.5MPa [39]. The pile tip bearing soil layer is non-collapsible loess with the void ratio *e*_0_ is 0.83 and the compression coefficient *α*_1−2_ is 0.14. According to the reference [34, 40], the relevant calculation parameters of loess in this region are listed in Table 1.

**Table 1:** Calculation parameters of loess.

Saturation gravity *γ*_sat_ (kN/m^3^)	Poisson’s ratio *μ*	Cohesion *c*(kPa)	Angle of internal friction *φ* (°)	Parameter *K*	Parameter *n*
18.9	0.4	25.4	23.5	19.3	0.733

**Fig 6:**
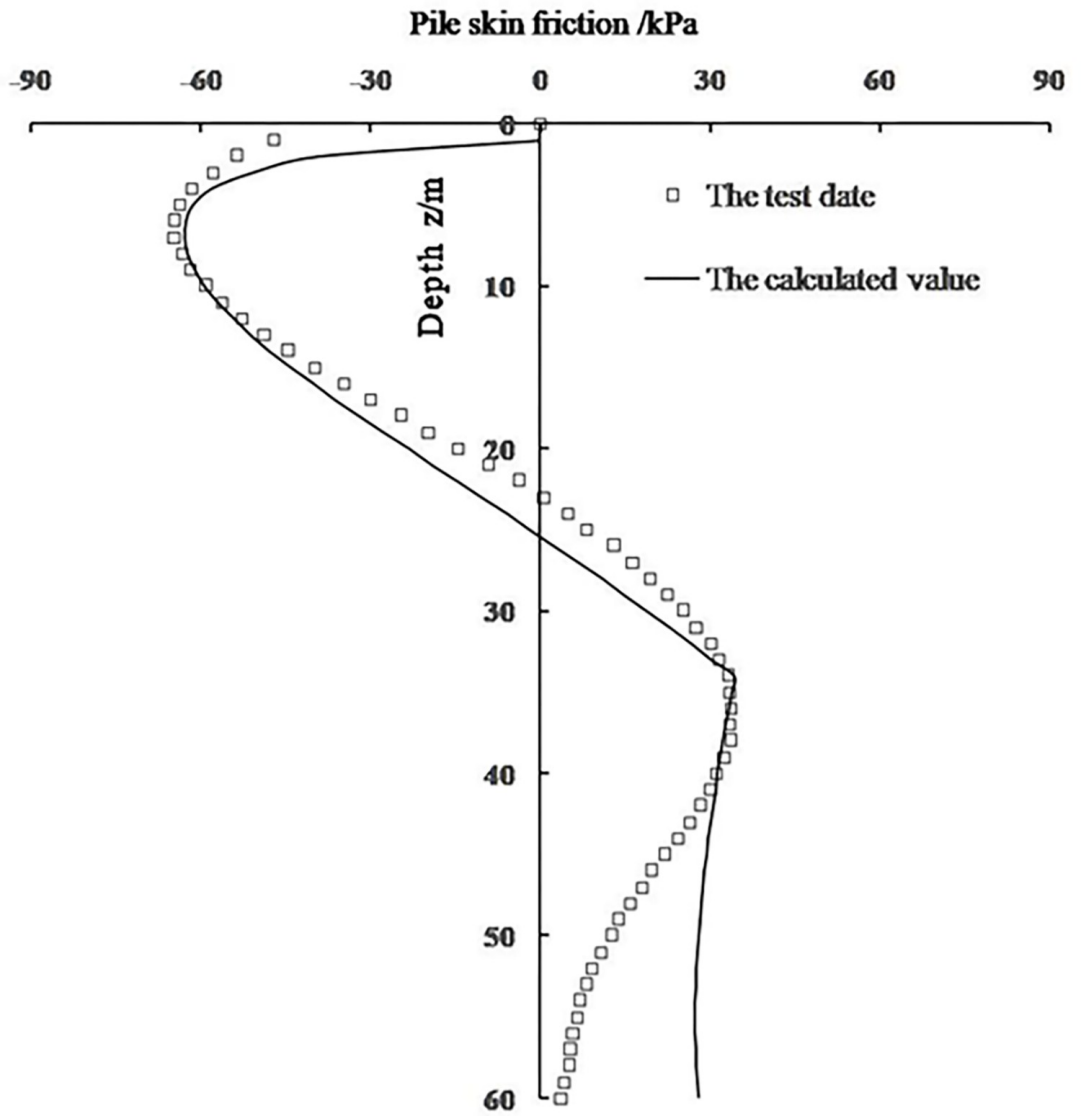
Comparison between the calculated value and test data.

The calculated value is compared with the test data, as shown in Fig 6. It can be seen that the calculated value is close to the test data of the filed immersion test. The ***calculated*** depth of the neutral point position is 24.5m, which is larger than the test depth (22m) with a relative error of 11.4%. The calculated subsidence of pile head is 7.5 mm, which is smaller than the test value (8.2 mm) with a relative error of 8.5%. The calculated value of pile negative skin resistance is consistent with the test value, the calculation deviation is mainly reflected in the calculation of positive shaft resistance, and the calculated results are larger than the test values. The deviation is due to that the subsidence of soil layer below the lower limit collapse depth is assumed to be 0, which causes the calculated value of pile-soil relative displacement within this soil layer to be larger than the actual value. After analyzing the influence of model parameters on the calculation results, it is found that the cohesion of loess is the greatest influencing factor. By comparing with the test data of pile foundation on-site immersion test, the calculation method proposed in this paper can realize the calculation of single pile bearing behavior in loess site.

## Conclusion

In this study, the calculation method of single pile bearing behavior under moistening deformation condition is established. The effectiveness of this method is verified by comparing with the test data that obtaining from a pile foundation on-site immersion test. The following conclusions can be drawn:

- The subsidence calculation method of soil layer at any depth is proposed. This method defines the collapse deformation calculation range of loess layers, and the equivalent calculation between the concentrated force of Boussinesq’s solution and the total self-weight collapse settlement.
- A new pile-soil load transfer constitutive model based on the strength and deformation of the soil around the pile is developed. This model takes into account the nonlinear shear deformation and ultimate shear strength of loess, and the pile skin friction under different relative displacement of pile-soil can be correctly reflect.
- The calculation model of single pile bearing behavior is established. This model is established according to the static balance of the pile element, and can calculate the pile axial force, the pile skin frictional, neutral point position and the settlement of a single pile.
- The calculation method is reasonable by comparison with the on-site immersion test. The calculation result show that the distribution rule of pile skin friction is similar to on-site test results, and the calculation error of neutral point position and pile settlement is within 10%.

## Supporting information

S1 File The relevant data in the manuscript. (DOCX)
