# Supplementary material for: Research on the bearing behavior of single pile in self-weight collapsible loess areas
Source: PLoS One. 2023 Aug 31;18(8):e0290878. doi: 10.1371/journal.pone.0290878 (PMC10470878; doi:10.1371/journal.pone.0290878)
Supplement: S1 File — (DOCX) [file pone.0290878.s001.docx]

**The relevant data in the manuscript**

| **Depth /m** | **The pile skin frictional** | |
| --- | --- | --- |
|  | **The test data/kPa** | **The calculated value/kPa** |
| 0 | 0 | 0 |
| 1 | -47.02 | -39.28 |
| 2 | -53.67 | -50.57 |
| 3 | -57.82 | -57.98 |
| 4 | -61.56 | -61.39 |
| 5 | -63.64 | -62.60 |
| 6 | -64.69 | -62.81 |
| 7 | -64.49 | -62.25 |
| 8 | -63.25 | -61.04 |
| 9 | -61.80 | -59.26 |
| 10 | -58.91 | -56.98 |
| 11 | -56.22 | -54.25 |
| 12 | -52.70 | -51.15 |
| 13 | -48.55 | -47.74 |
| 14 | -44.41 | -44.06 |
| 15 | -39.85 | -40.17 |
| 16 | -34.68 | -36.12 |
| 17 | -29.91 | -31.94 |
| 18 | -24.52 | -27.68 |
| 19 | -19.55 | -23.35 |
| 20 | -14.58 | -19.00 |
| 21 | -9.19 | -14.64 |
| 22 | -3.81 | -10.30 |
| 23 | 0.75 | -5.99 |
| 24 | 4.90 | -1.71 |
| 25 | 8.21 | 2.51 |
| 26 | 13.18 | 6.68 |
| 27 | 16.49 | 10.78 |
| 28 | 19.39 | 14.82 |
| 29 | 22.50 | 18.81 |
| 30 | 25.18 | 22.73 |
| 31 | 27.67 | 26.59 |
| 32 | 30.15 | 30.41 |
| 33 | 31.60 | 34.19 |
| 34 | 33.25 | 33.74 |
| 35 | 33.45 | 33.25 |
| 36 | 33.65 | 32.76 |
| 37 | 33.44 | 32.28 |
| 38 | 33.64 | 31.81 |
| 39 | 32.59 | 31.35 |
| 40 | 31.13 | 30.91 |
| 41 | 30.30 | 30.49 |
| 42 | 28.21 | 30.08 |
| 43 | 26.34 | 29.69 |
| 44 | 24.26 | 29.33 |
| 45 | 21.97 | 28.99 |
| 46 | 19.68 | 28.68 |
| 47 | 18.01 | 28.39 |
| 48 | 15.93 | 28.14 |
| 49 | 13.85 | 27.92 |
| 50 | 12.60 | 27.73 |
| 51 | 10.73 | 27.57 |
| 52 | 9.06 | 27.46 |
| 53 | 8.22 | 27.38 |
| 54 | 6.97 | 27.35 |
| 55 | 6.55 | 27.35 |
| 56 | 5.92 | 27.41 |
| 57 | 5.50 | 27.51 |
| 58 | 5.08 | 27.66 |
| 59 | 4.24 | 27.87 |
| 60 | 3.82 | 28.13 |
